# Effects and safety of Salvia miltiorrhiza on the improvement of renal function, inflammatory factors, and vascular endothelium in patients with diabetic kidney disease: a meta-analysis and systematic review

**DOI:** 10.3389/fphar.2025.1556368

**Published:** 2025-09-12

**Authors:** Shuyu Zheng, Meng Zhang, Wenkuan Wang, Qian Zhang, Ning Zhang

**Affiliations:** ^1^ Graduate School of Beijing University of Chinese Medicine, Beijing, China; ^2^ Department of Nephrology and Endocrinology, Wangjing Hospital, China Academy of Traditional Chinese Medicine, Beijing, China

**Keywords:** traditional Chinese medicine, Salvia miltiorrhiza (SM), Salvia miltiorrhiza preparations, diabetic kidney disease, meta-analysis

## Abstract

**Objective:**

To elucidate the efficacy and safety of Radix et rhizoma Salvia miltiorrhiza (SM) in the treatment of Diabetic Kidney Disease (DKD), and to provide a rationale and scientific reference for the use of SM preparations in the treatment of DKD. This study is the first systematic evaluation and Meta-analysis focusing exclusively on the use of SM as a single agent in the treatment of DKD.

**Methods:**

A comprehensive search was conducted in PubMed, Web of Science, Cochrane Library, Elsevier Science Direct, CNKI, Wanfang, and VIP databases, covering the timeframe from the inception of the journals to May 2025. The search was restricted to randomized controlled trials conducted within the past decade that investigated the use of SM/SM preparations as a treatment for DKD. The control group received conventional interventions, while the intervention group received SM/SM preparations. Endnote 20 and Excel were employed for literature management and data organization, and Revman 5.3 and Stata 18 software were used for the analyses.

**Results:**

This study involved 21 RCTs with 1970 participants. The results demonstrated that SM preparations led to reductions in serum creatinine (Scr), blood urea nitrogen (BUN), urinary albumin excretion rate (UAER), 24-h urinary total protein (24 h-utp), C-reactive protein (CRP), tumor necrosis factor-alpha (TNF-α), interleukin-6 (IL-6), and endothelin-1 (ET-1) levels among patients with DKD (*P* < 0.05). Moreover, these preparations elevated flow-mediated vasodilation (FDM), showcasing their clinical effectiveness over the control group (*P* < 0.05). Notably, the safety profile remained sound, with no significant differences in adverse event rates between the two groups (*P* > 0.05).

**Conclusion:**

These results indicate that SM preparations could considerably improve renal and vascular endothelial function while simultaneously decreasing harmful inflammatory markers in patients with DKD, which allow it serve as a safe and effective therapeutic option.

**Systematic Review Registration:**

https://www.crd.york.ac.uk/PROSPERO/#recordDetails, identifier CRD42024623452.

## 1 Background

Diabetic kidney disease (DKD) is a complication of diabetes mellitus characterized by microvascular damage. Clinically, it presents as a persistent increase in albuminuria excretion and/or a progressive decline in glomerular filtration rate (GFR) (St and anton, 2014). DKD is a leading cause of end-stage renal disease (ESRD), accounting for 30%–50% of ESRD cases globally (Ruiz-Ortega et al., 2020). In China, it has become the predominant cause of ESRD among middle-aged and elderly individuals (Expert Group of the Chinese Society of Nephrology, 2021).

Although existing treatments such as glycaemic control, blood pressure management, and renin-angiotensin-aldosterone system (RAAS) inhibitors have had some success in slowing the progression of DKD, problems such as limited therapeutic efficacy and obvious side effects still exist. Therefore, there is an urgent need to find extra treatment strategies (Sugahara et al., 2021). Traditional Chinese medicine (TCM) has demonstrated its value in preventing and treating DKD, with studies showing significant advantages in improving renal function, delaying progression to end-stage renal disease, and enhancing the quality of life for patients in the early and middle stages of the disease. Furthermore, studies have demonstrated that combining TCM treatment with dialysis can reduce patient mortality (China Association of Chinese Medicine et al., 2024; Tang et al., 2021).

The root and rhizome of *Salvia miltiorrhiza* Bunge (Lamiaceae), known as Radix et Rhizoma Salvia miltiorrhiza (SM) or Danshen is one of key botanical drugs of TCM, which is included one of the five core medicine in 5,901 TCM prescriptions (Guo et al., 2021). Our previous study found that SM was the second most frequently used herb after astragalus in 66 randomized controlled trials of Chinese botanical drugs for DKD (Zheng et al., 2024). SM is a classical TCM herb widely used to promote blood circulation and remove blood stasis, particularly for conditions associated with microvascular obstruction. In TCM theory, DKD falls under the category of “Xiaoke”, with “blood stasis obstructing meridians” being a key pathological mechanism. SM is believed to alleviate DKD pathology by enhancing microcirculation and resolving stasis.

Modern pharmacological research has identified multiple bioactive metabolites in SM, primarily including water-soluble phenolic acids such as salvianolic acid A and B and lipophilic diterpenoids such as tanshinone IIA. These constituents exhibit anti-inflammatory, antioxidant, and anti-fibrotic effects. Salvianolic acid B and tanshinone IIA have been shown to synergistically improve renal function and attenuate kidney injury in early DKD rats by modulating the PI3K/Akt/NF-κB signaling pathway (Hou et al., 2017). Moreover, salvianolic acid A can protect renal function by mitigating oxidative stress by inhibiting the AGE-RAGE signaling axis (Zeng et al., 2024).

Alongside efficacy, ensuring the consistency and controllability of SM’s medicinal quality has become a critical focus in modernizing TCM. Previous studies have used mathematical models to elucidate the relationship between secondary metabolite accumulation and plant growth status, which offers a theoretical basis for standardizing bioactive compound formation (Wang et al., 2022). Additionally, the combined use of high-performance liquid chromatography with diode-array detection (HPLC-DAD) and near-infrared spectroscopy (NIR), integrated with chemometric approaches, has enabled the development of a comprehensive quality evaluation system balancing qualitative and quantitative assessments (Li et al., 2022). These studies provide a robust foundation for standardizing SM-based clinical preparations.

Currently, the major clinical formulations of SM include SM injection, tanshinone IIA sodium sulfonate injection, and Salvia divinorum polyphenate injection, all of which are derived from specific SM active ingredients. SM injection is rich in water-soluble phenolic acids and is widely used to improve microcirculation, inhibit platelet aggregation, and suppress inflammation, especially in cardiovascular and diabetic complications (Yu et al., 2012; Shen et al., 2023). Tanshinone IIA sodium sulfonate injection, a water-soluble derivative synthesized from the lipophilic metabolites tanshinone IIA. It possesses potent antioxidant and anti-fibrotic activities and has been shown to significantly ameliorate renal hypertrophy and 24-h urinary protein excretion in DKD rats (Xiong et al., 2014). Salvia divinorum polyphenate, obtained by concentrating the phenolic metabolites of SM, demonstrates potential in retarding DKD progression through mechanisms including inhibition of platelet aggregation, antioxidant effects, and protection of vascular endothelial function (Kim et al., 2009).

SM preparations is often used in therapeutic procedures, but there is still limited systematic evaluation of clinical studies on SM preparations alone. Therefore, it is essential to conduct a thorough and systematic review of the efficacy of SM in treating DKD. This study performed a meta-analysis and systematic review to collect and analyze data from relevant randomized controlled trials (RCTs), aiming to clarify the effects and safety of SM on improving renal function, reducing inflammatory factors and enhancing vascular endothelial function in patients with DKD. The findings of this study will provide an evidence-based foundation and scientific reference for the use of SM in treating DKD.

## 2 Methods

### 2.1 Registration

The meta-analysis was registered with the International Prospective Register of Systematic Reviews (PROSPERO) under the registration number CRD42024623452. We followed the Preferred Reporting Items for Systematic Reviews and Meta-Analyses (PRISMA), its protocols, and the PRISMA-extension statementfor meta-analysis to report the current results (Hutton et al., 2015). For a more thorough understanding, please refer to the appendix PRISMA checklist.

### 2.2 Search methods

We conducted a systematic search across multiple databases, including PubMed, Web of Science, Cochrane Library, Elsevier Science Direct, CNKI, Wanfang, and VIP databases, covering the timeframe from the inception of the journals to November 2024. Our Chinese search terms were precisely crafted: (diabetic nephropathy + diabetic kidney disease + Xiaoke nephropathy), (danshen + danshen preparation), and (randomized controlled trial + clinical trial). For the English literature, we employed strategic search terms: (diabetic kidney disease and diabetic nephropathy), (Salvia miltiorrhizae and Danshen), and (Randomized Controlled Trial and Clinical Trial and Intervention Study and Clinical Study). For further details, please refer to the specific search strategy outlined in Supplementary Appendix Table 1.

We screened the reference lists of related articles and reviews to identify additional studies that could enhance our research. Both the title/abstract and full-text screenings were conducted independently by two researchers (Zheng Shuyu and Zhang Meng). In instances of disagreement between them, we ensured accuracy and reliability by involving a third researcher, Zhang Ning, to make the final decision.

### 2.3 Selection criteria

We focused on randomized controlled trials that investigated the effectiveness of SM in treating DKD, ensuring no limitations regarding the language of the studies, geographic location, or duration.

Our inclusion criteria are as follows:

a. Only randomized controlled trials were considered.
b. Participants included adults (aged 18 years and above) with a clinically confirmed diagnosis of DKD.
c. Treatment groups included those receiving SM/SM preparations, contrasted against a control group that did not receive such treatments.
d. The studies had to report at least one of the following critical outcomes: renal function indices including serum creatinine (SCR), blood urea nitrogen (BUN), urinary albumin excretion rate (UAER), or 24-h urine protein (24 h-utp), inflammatory markers including C-reactive protein (CRP), tumor necrosis factor-alpha (TNF-a), and interleukin-6 (IL-6)), or vascular endothelial indicators including endothelin-1 (ET-1), and flow-mediated dilation (FMD).

We excluded studies based on these criteria:

a. Non-randomized studies, retrospective analyses, animal experiments, and review articles were not considered.
b. Participants with renal impairment from causes other than DKD, as well as those experiencing acute or subacute renal failure, were excluded.
c. Intervention groups employing treatments beyond SM/SM preparations—whether that includes additional drugs or metabolite SM combinations—were also excluded.
d. Studies with flawed data, incomplete outcome measurements, or any data that could not be sourced from the original authors were eliminated.
e. Duplicate publications were disregarded.

### 2.4 Data extraction

To ensure the highest quality and accuracy in our review, two researchers (Wang Wenkuan and Zhang Qian) extracted data from studies that met our inclusion criteria. In case of differing opinions, we facilitated a consensus among all authors. The extracted data included the following vital components:

a. Publication Information: This essential data encompasses the title, first author, and year of publication.
b. Study Characteristics: study design and treatment period.
c. Subject Characteristics: sample size, age, gender, duration of DKD, and DKD stage.
d. Intervention: Both the control and treatment groups’ interventions including the specific drugs administered, drug manufactures, dosages, modes of administration, and frequencies.
e. Outcome Indicators: primary outcome indicators including renal function indices (SCR, UAER, BUN, 24 h-utp), inflammatory markers (CRP, TNF-α, IL-6), and vascular endothelial indicators (ET-1, FMD). Secondary outcome indicators covering efficacy and adverse events.

In cases where the published data raised questions or appeared incomplete, we proactively engaged with the authors to request the necessary information, reinforcing our dedication to data integrity and thoroughness.

All SM preparations were commercially procured from certified manufacturers, whose production processes are regulated under the Pharmacopoeia of the People’s Republic of China (2020 edition) (National Pharmacopoeia Committee, 2020). This regulatory framework mandates minimum content thresholds for key metabolites, ensuring baseline pharmacological consistency. See Supplementary Appendix Table 2 for details of drug information.

### 2.5 Data coding and management

Endnote 20.01 was employed to manage the literature, complemented by Excel and SPSS for data management. Two researchers (Zheng Shuyu and Zhang Meng) independently assessed the quality of the RCT studies using the comprehensive risk of bias assessment tool from Cochrane, conducting their assessments in sync and cross-verifying their results. Our assessment included seven components: the creation of randomized sequences, allocation concealment, blinding of both participants and researchers, completeness of outcome data, reporting bias, and other potential biases. The methodological quality of each study was systematically categorized into three distinct levels: high risk, low risk, and uncertain bias. Data processing adhered to the ConPhyMP framework, and the completed checklists are included in the supplementary materials (Heinrich et al., 2022).

### 2.6 Statistical analysis

We used Revman 5.3, and Stata 18.0 for the statistical data analysis. Effect analysis statistics were OR RR for dichotomous variables and MD or SMD for continuous variables, using 95% confidence intervals (CI), Cochrane Q statistic, and *I* ^*2*^ test for detecting heterogeneity. If *P* ≥ 0.05 and *I* ^*2*^ < 50%, analyses were conducted using a fixed-effects model. If *P* < 0.05 and *I* ^*2*^ ≥ 50%, analyses were performed using a random effects model (Higgins and Thompson, 2002). *α* = 0.05 was used as the meta-analysis test level. For outcomes with significant heterogeneity, sensitivity analyses were performed using the one-by-one exclusion method. Subgroup analyses will be performed on the different outcome indicators, and the subgroups will be grouped on treatment period (<4 weeks, ≥4 weeks), drug dosage of the treatment group (SM injection, Tanshinone IIA sodium sulfonate injection, Salvia divinorum polyphenate). Publication bias was assessed using funnel plots, and Egger’s test. Studies at high risk of bias were corrected and evaluated using the trim and fill method.

## 3 Results

**FIGURE 1:**
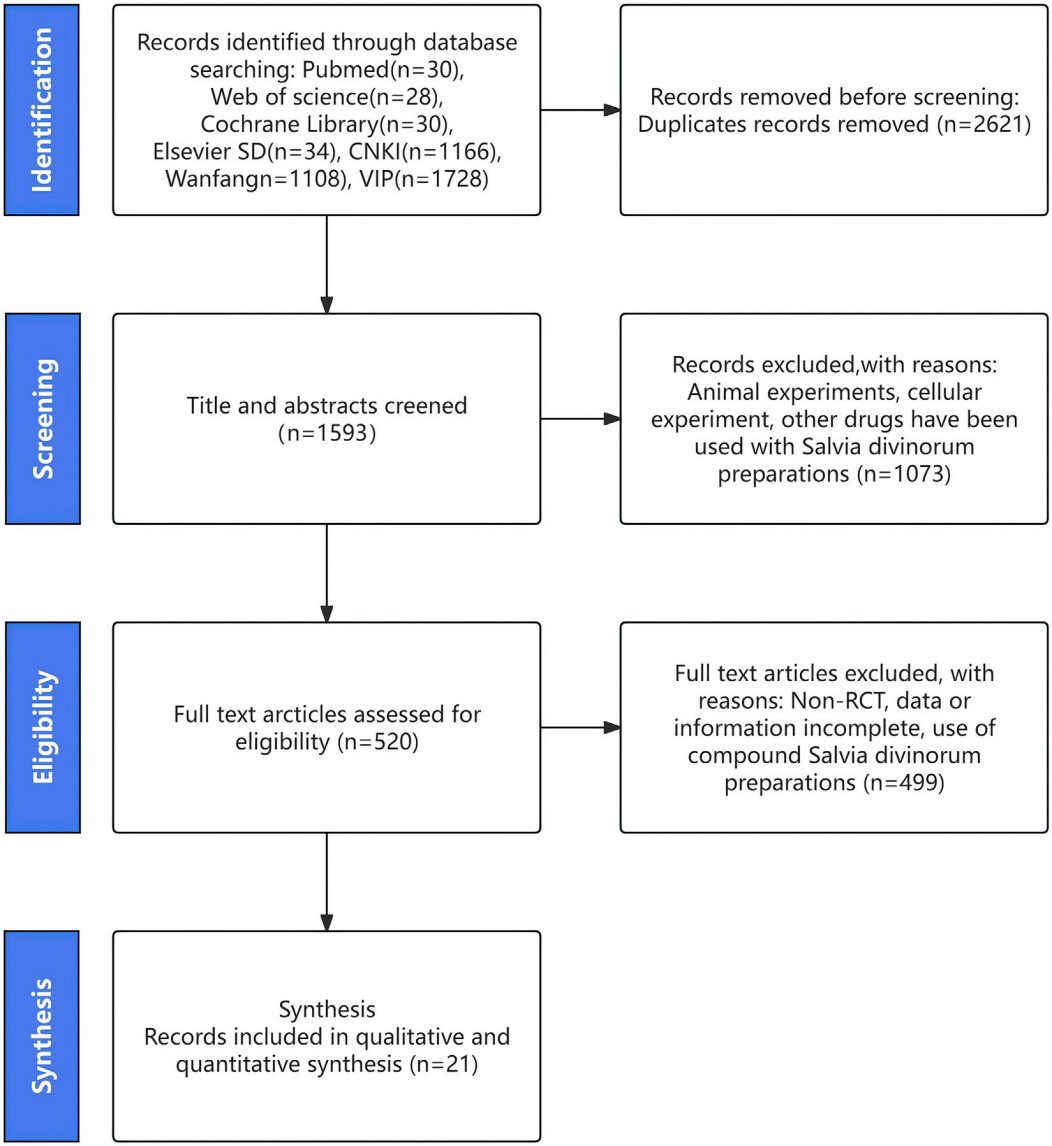
Flow plot.

After searching the database, we identified 4,142 potentially eligible studies. We then reviewed the full text of 520 of these studies after removing duplicates and screening the titles and abstracts. Ultimately, we included 21 studies in our analysis, which we selected by comparing them against the inclusionandextraction criteria (Hu et al., 2006; Hu and He, 2007; Liu et al., 2008; Leng, 2011; Bowen et al., 2012; Liu et al., 2014; Lu et al., 2014; Yusuf and Hongli, 2015; Hu et al., 2016; Chen et al., 2018; Fu and Shao, 2018; Qiao, 2018; Wang et al., 2018; Jun et al., 2018; He and Zhu, 2019; Hong, 2020; Li et al., 2020; He et al., 2021; Wang et al., 2021; Zhao, 2025). The specific pathway for screening is detailed in Figure 1.

### 3.1 Study characteristics

The studies included in this review span from 2002 to 2025, comprising a sample of 1970 patients, with 988 in the treatment group and 982 in the control group. All research was conducted in China. The mean ages of participants in the treatment group varied from 48.4 to 68.45 years, while the control group’s mean ages ranged from 45.32 to 68.82 years. Treatment durations were comprehensive, ranging from 2 weeks to an impressive 52 weeks. Patients were classified according to the DN Mogensen staging system, with representations from stages III to V. Interventions in the control group included conventional therapy, ACEI/ARB drugs, insulin, prostaglandins, haemodialysis, and alfacalcitol. The treatment groups focused on the integration of SM preparations into the control treatments. Interventions for the treatment groups featured a range of options, such as SM injections, tanshinone IIA sodium sulfonate injections and Salvia divinorum polyphenate injection. See Supplementary Appendix Table 3 for a list of characteristics of the included studies, Supplementary Appendix Table 4 for details of the interventions included in the studies.

### 3.2 Study assessment

**FIGURE 2:**
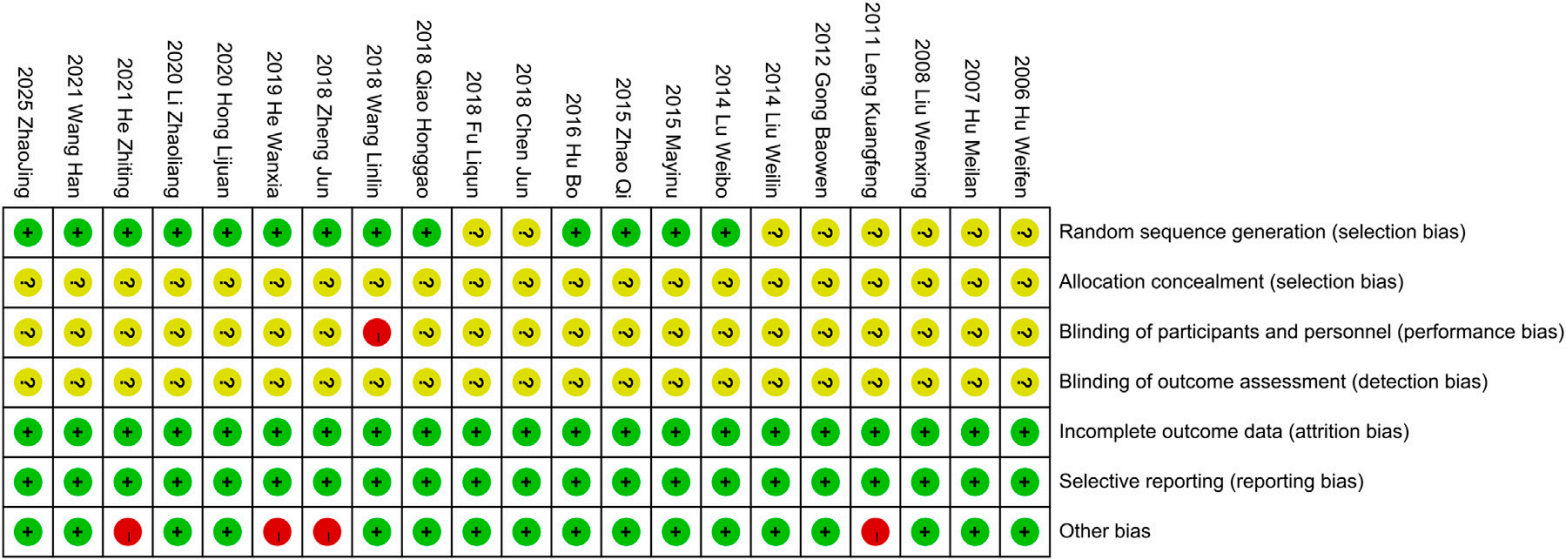
Risk of bias summary.

**FIGURE 3:**
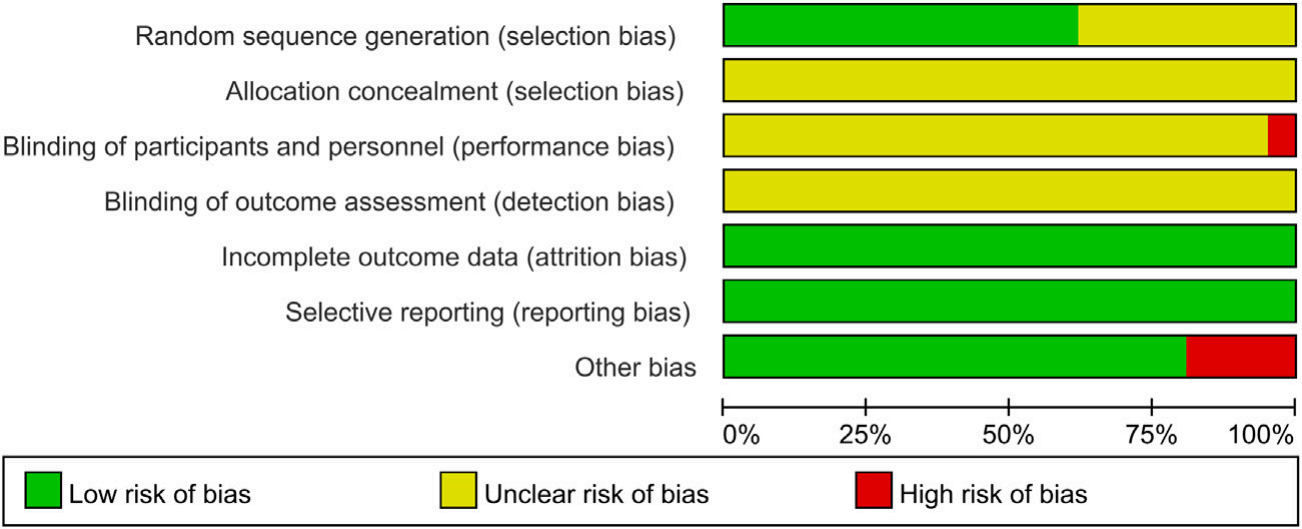
Risk of bias graph.

A thorough quality assessment of the literature was conducted using the Cochrane Risk Assessment Tool. Among the 21 studies included, 13 employed the randomized table of numbers method, earning a commendable low-risk rating and the remaining studies did not specify their randomization techniques, which resulted in an unknown risk designation. None of the literature clearly outlined the allocation methods, reinforcing the unknown risk evaluation in that area. Notably, Wang et al. (2018) indicated the use of single blinding, which warranted a high-risk rating. The other studies, lacking any mention of blinding, were subsequently categorized as unknown risk. All the studies provided clear outcome data justifying a low-risk rating. All studies were not registered and selective reporting could not be judged and was evaluated as an unknown risk. Some studies had potential other bias due to inclusion of patients with comorbid other chronic diseases or end-stage renal disease, and the studies of Leng, (2011), Jun et al. (2018), He and Zhu, (2019), He et al. (2021) were rated as high risk of bias. For a more detail, please refer to Figures 2, 3.

In addition, we systematically evaluated the quality of evidence for the primary outcomes using the GRADE method. According to the assessment, the quality of evidence for most of the outcome indicators was of moderate or low grade. Due to the imperfect reporting of hidden allocation and blinding in some studies, the presence of unstable confidence intervals for the primary outcome indicators and the lack of clearly stated registration information, the relevant dimensions were considered to be downgraded. For a more detail, please refer to Supplementary Appendix Table 5.

### 3.3 Kidney function

**FIGURE 4:**
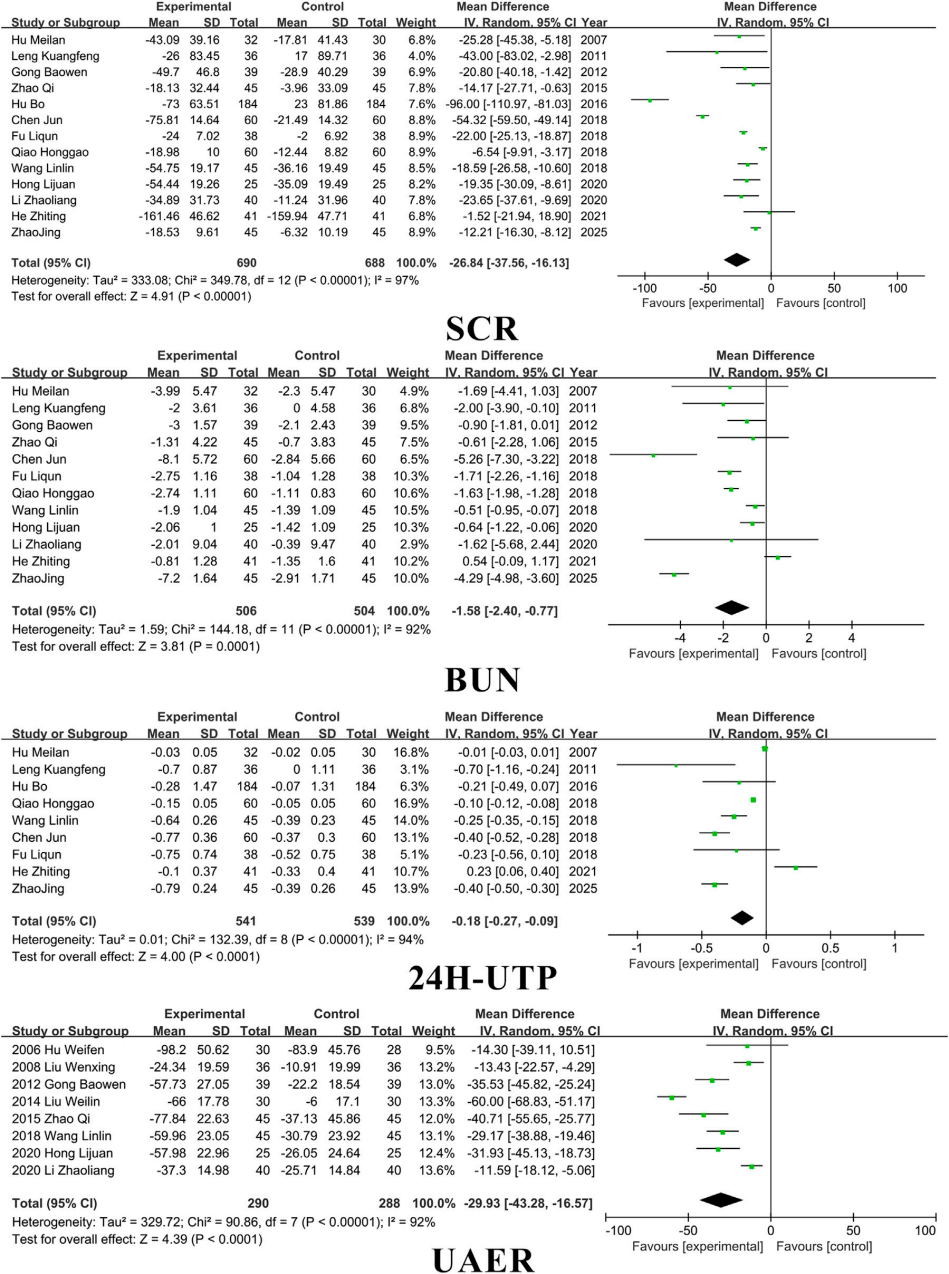
Kidney function.

Fourteen studies included SCR, thirteen studies included BUN, eight studies included UAER, and ten studies included 24 h-UTP. SM preparations demonstrated a significantly higher efficacy in reducing SCR, BUN, UAER, and 24 h-UTP levels in patients with DKD when compared to the control group. (SCR: MD =-26.84, 95% CI [-37.56, −16.13], *I* ^*2*^= 97%, *P* < 0.00001; BUN: MD = −1.58, 95% CI [-2.40, −0.77], *I* ^*2*^= 97%, *P* < 0.0001; UAER: MD =-0.18, 95% CI [-0.27, −0.09], *I* ^*2*^= 94%, *P* < 0.001; 24 h-utp: MD =-29.93, 95% CI [-43.28, −16.57], *I* ^*2*^= 92%, *P* < 0.0001). For detailed insights, please refer to Figure 4.

**FIGURE 5:**
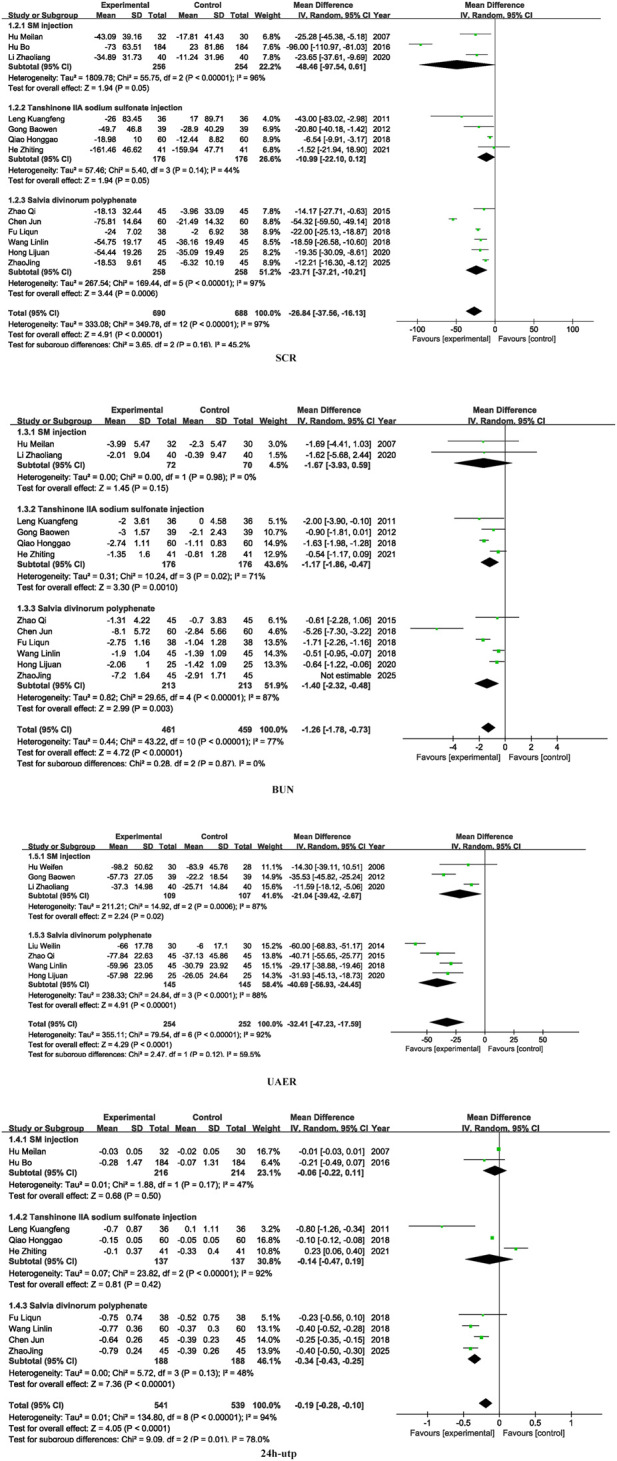
Kidney function drug dosage subgroup.

Regarding subgroup analyses of renal function, the overall effect of SCR was significant (*P* < 0.0001), Subgroup analysis based on different dosages of SM preparations revealed no statistically significant differences in SCR (*P* = 0.16), BUN (*P* = 0.87), or UAER (*P* = 0.87), suggesting that formulation type may not be the primary source of heterogeneity for these outcomes. However, a significant subgroup difference was observed for 24 h-UTP (*P* = 0.01), indicating that the dosages of SM preparations may contribute to heterogeneity in this particular outcome and warrants further investigation into pharmacokinetic differences among formulations. For detailed insights, please refer to Figure 5.

**FIGURE 6:**
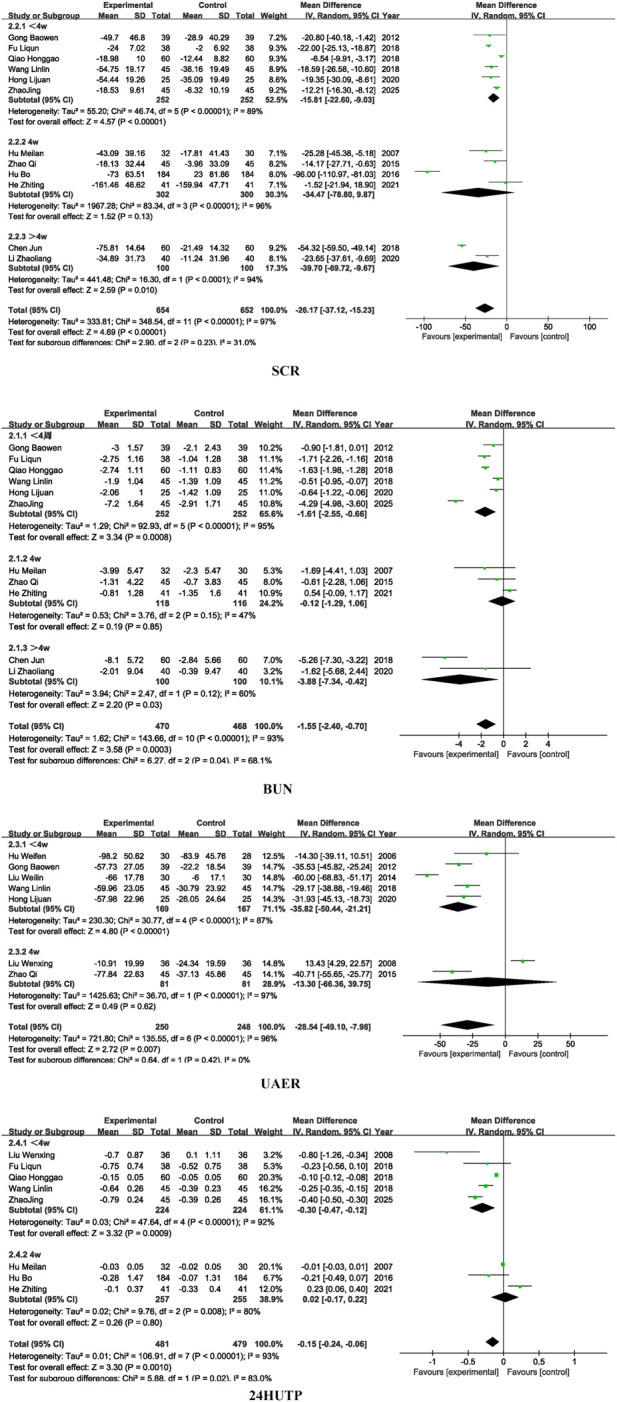
Kidney function treatment period subgroup.

Similarly, subgroup analysis based on treatment duration showed no significant intergroup differences for SCR (*P* = 0.23) and UAER (*P* = 0.42), implying that treatment duration is unlikely to be the main source of heterogeneity for these indicators. In contrast, statistically significant subgroup differences were observed for BUN (*P* = 0.04) and 24 h-UTP (*P* = 0.02), suggesting that variation in treatment duration may account for a substantial portion of heterogeneity in these outcomes. For detailed insights, please refer to Figure 6.

The observed heterogeneity in 24 h-UTP related to formulation type may be attributed to the differences in chemical composition, bioavailability, and pharmacokinetics among various SM formulations. These variations can influence the extent to which each formulation reduces proteinuria, a sensitive marker of glomerular barrier integrity (Zhang et al., 2022). Additionally, the heterogeneity observed in BUN and 24 h-UTP across different treatment durations likely reflects the time-dependent nature of SM’s therapeutic effects. Short-term interventions may be insufficient to elicit measurable changes in these indicators, which are influenced by both glomerular and tubular function.

### 3.4 Inflammatory factors

**FIGURE 7:**
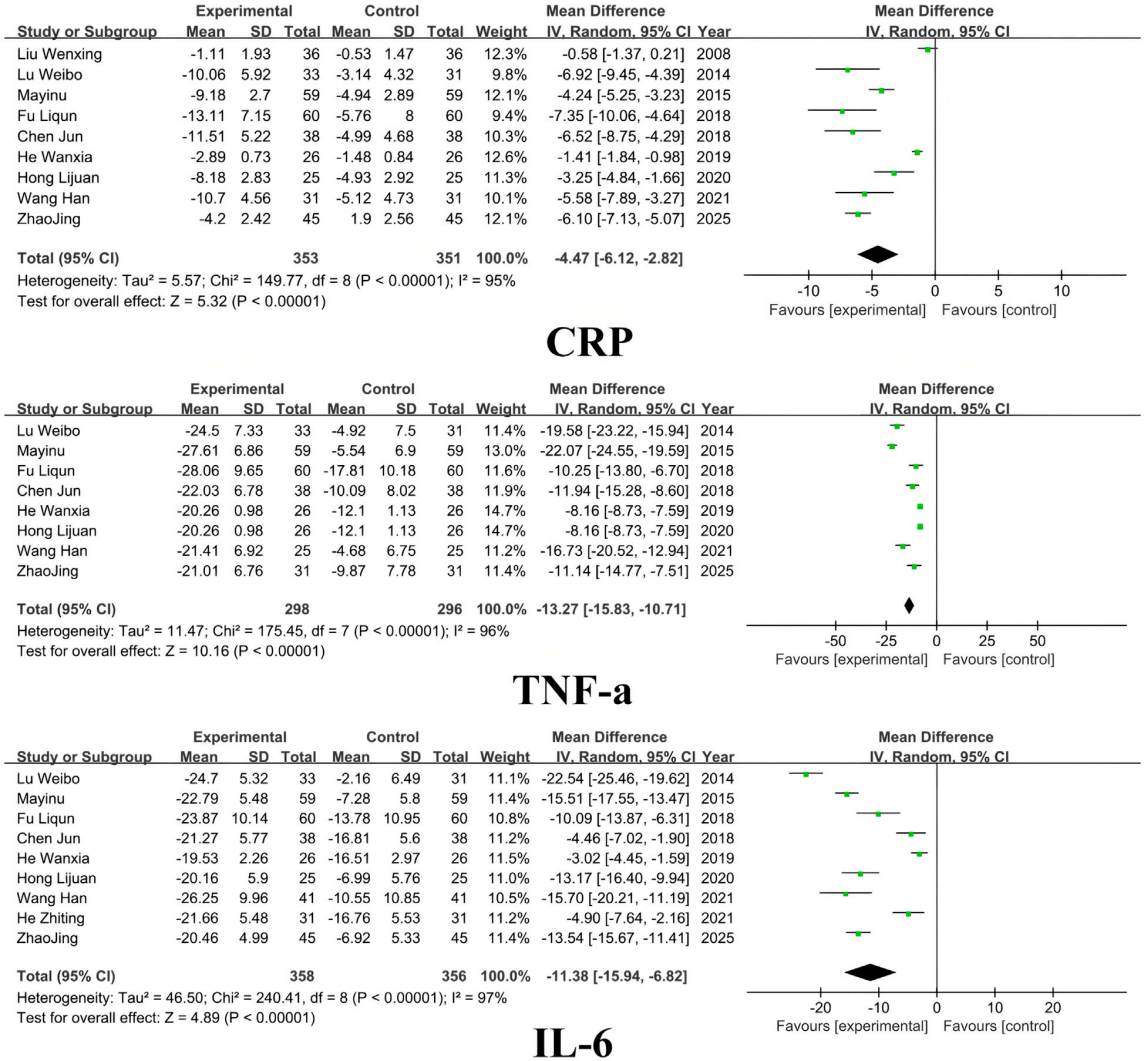
Inflammatory factors.

Nine studies included CRP indexes, eight studies included TNF-a, and nine studies included IL-6. The results showed that SM preparations group was significantly more effective in lowering CRP, TNF-a, and IL-6 in patients with DKD than in the control group (CRP: MD =-4.47, 95% CI [-6.12, −2.82], *I* ^*2*^= 95%, *P* < 0.00001; TNF-a: MD = −13.27, 95% CI [-15.83, -10.71], *I* ^*2*^= 96%, *P* < 0.00001; IL-6: MD =-11.38, 95% CI [-15.94, -6.82], *I* ^*2*^= 97%, *P* < 0.00001). For detailed insights, please refer to Figure 7.

### 3.5 Vascular endothelial function

**FIGURE 8:**
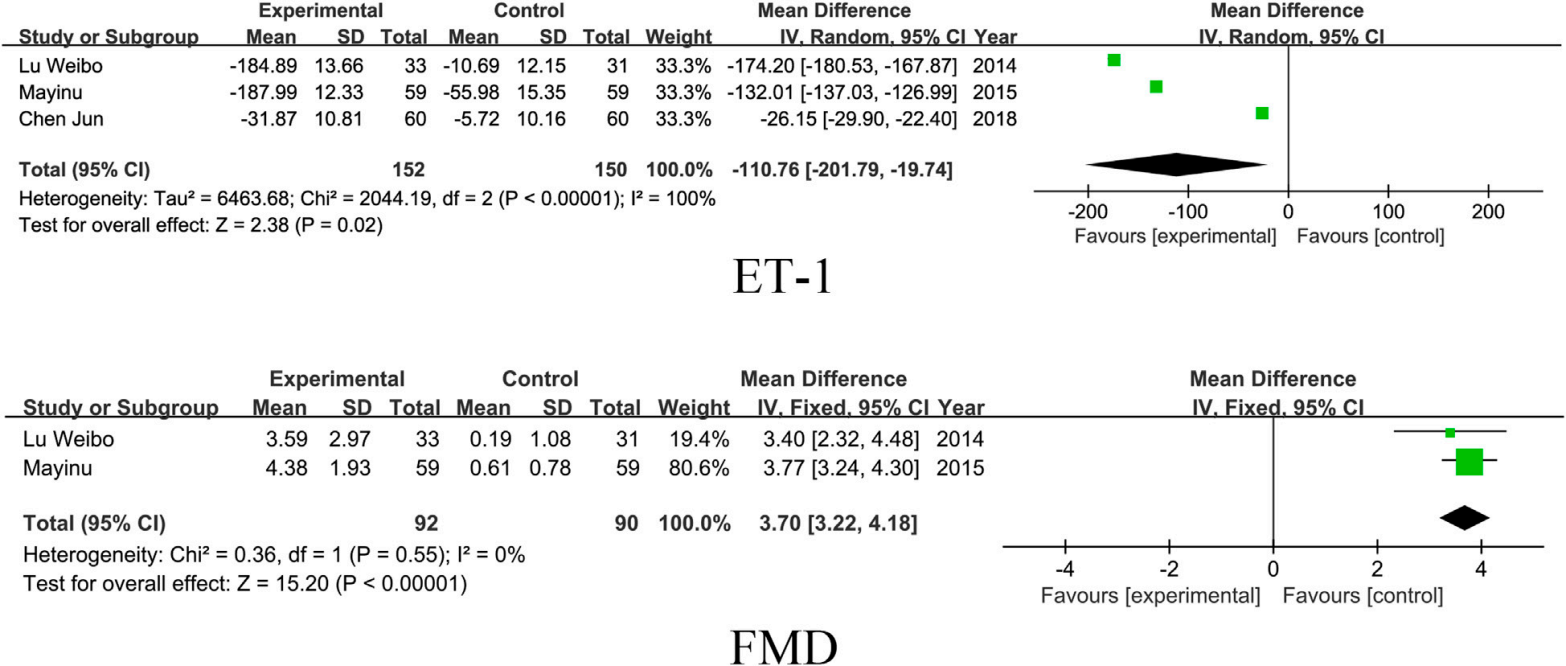
Vascular endothelial function.

Three studies included the ET-1 index and two studies included FMD. The results showed that SM preparations group reduced ET-1 and improved FMD significantly better than the control group in patients with DKD (ET-1: MD = -110.76, 95% CI [-201.79, −19.74], I^2^= 100%, P =0.02; FMD: MD = 3.70, 95% CI [3.22, 4.18], I^2^= 0%, P < 0.00001). For detailed insights, please refer to Figure 8.

### 3.6 Efficacy

**FIGURE 9:**
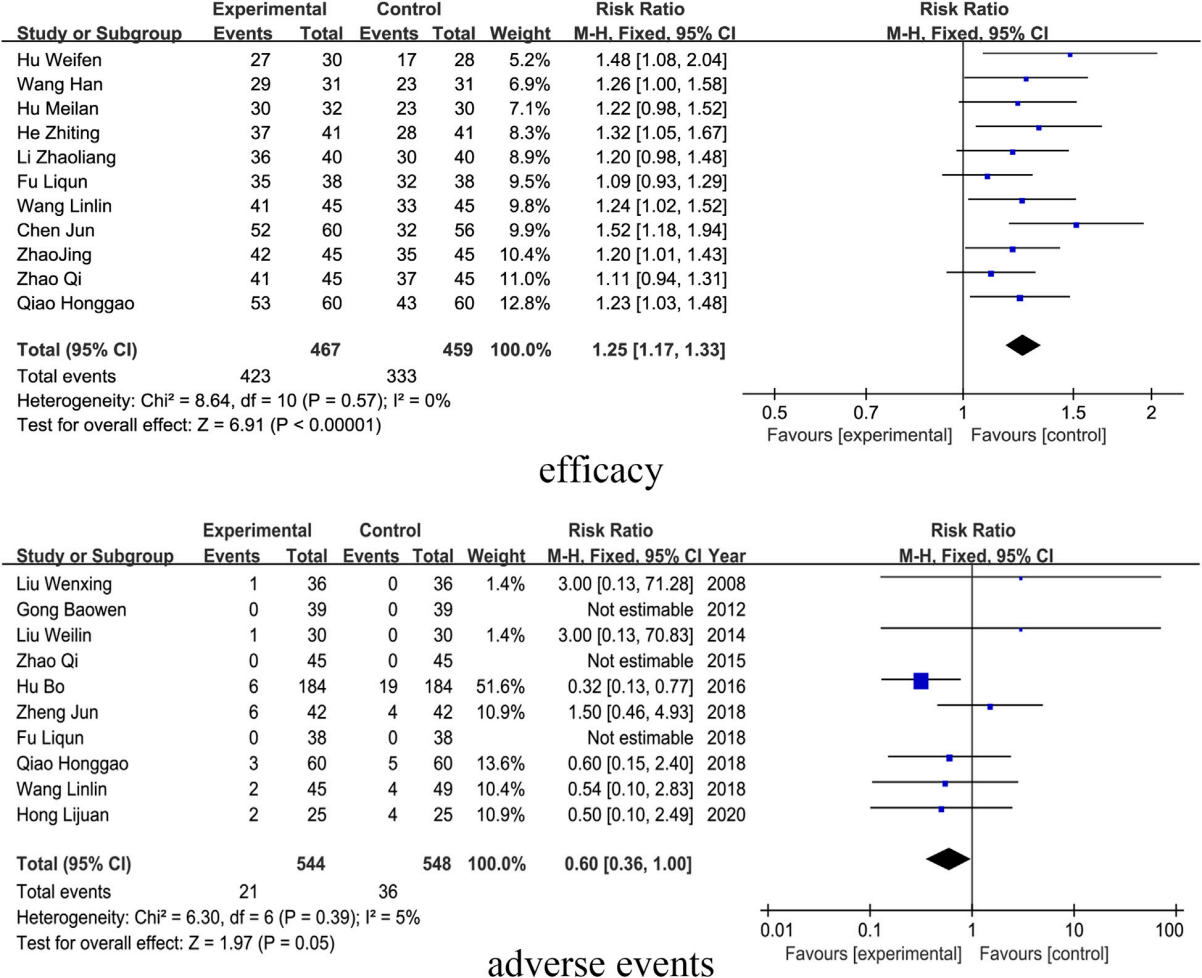
Dichotomous variable.

Elven studies included outcome indicators of clinical efficacy. The results showed that the SM preparations group improved the clinical efficacy of patients with DKD significantly more than that of the control group (RR = 1.25, 95% CI [1.17, 1.33], *I* ^*2*^= 0%, *P* < 0.0001). For detailed insights, please refer to Figure 9.

### 3.7 Adverse events

Ten studies reported on adverse events, which are detailed in Supplementary Appendix Table 6. The results indicated that the incidence of adverse events in patients with DKD treated with SM preparations was not significantly different from that in the control group (RR= 0.60, 95% CI [0.36, 1.00], *I* ^*2*^= 0%, *P* = 0.05). For detailed insights, please refer to Figure 9.

### 3.8 Sensitivity analysis and publication bias

Sensitivity analyses were assessed using a drop-by-study approach, and the results showed that the change in the combined effect sizes was not significant after dropping any of the studies, indicating that the overall analysis was robust.

**FIGURE 10:**
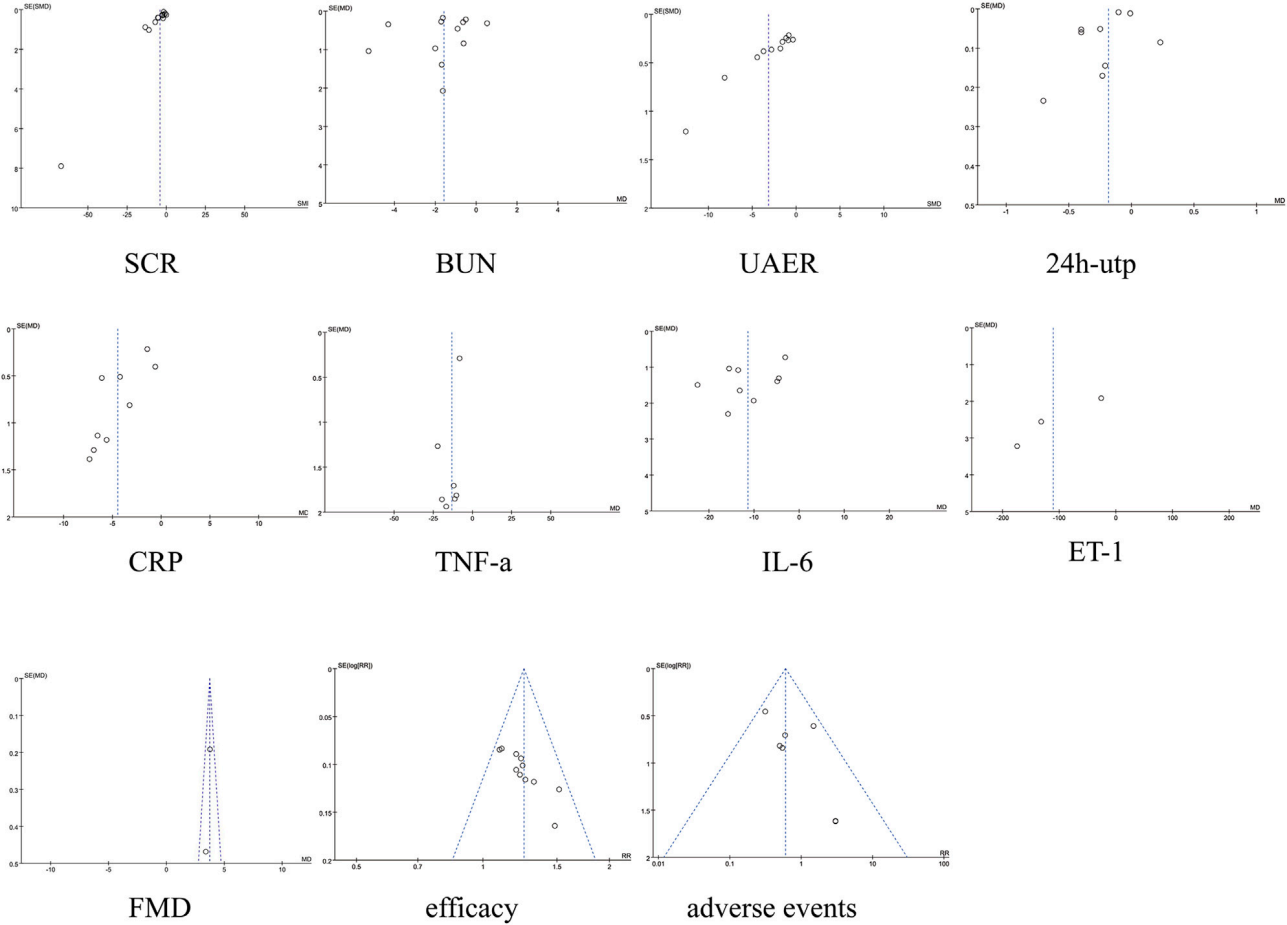
Funnel plots.

**FIGURE 11:**
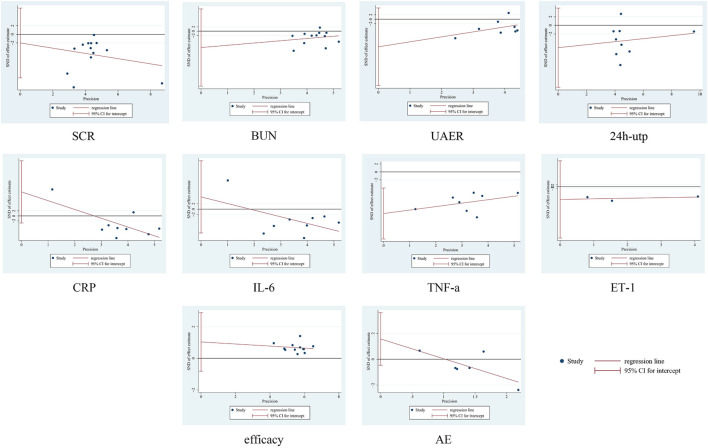
Egger plots.

No significant asymmetry was seen in the funnel plot, for detailed insights, please refer to Figure 10. By Egger’s test, the results showed that there was publication bias for TNF-a (*P* = 0.007), and there was no publication bias for the rest of the results (*P* > 0.05). See Figure 10. To further assess the effect of bias on the combined effect value, correction was applied by the cut-and-patch method. The analysis showed that three studies were possibly missing, and the total number of studies increased to 11 after repair. The random effects model was used to recalculate the combined effect size after repair, and the result was MD = −3.639 (95% CI [-4.912, −2.366], *P* < 0.001), and the effect value was still statistically significant, suggesting that the results of the present study were still robust. For detailed insights, please refer to Figure 11.

## 4 Discussion

China currently has the largest diabetic population in the world, and DKD, a common and serious complication, remains a leading cause of death among these patients. Timely and suitable use of TCM can significantly enhance the wellbeing and longevity of patients with DKD, providing a valuable complement to their treatment.

In TCM, blood stasis significantly influences the development of DKD. As a result, TCM frequently emphasizes the importance of enhancing kidney function and improving blood circulation as key treatment strategies. According to *Bencao Zhengyi*, SM is recognized for its remarkable ability to penetrate the internal organs, effectively removing blood stasis while also providing external benefits to the joints and enhancing the flow within veins and channels. SM excels in activating blood circulation, alleviating blood stasis, promoting menstruation, relieving various types of pain, soothing the heart, alleviating stress, cooling the blood, and addressing skin carbuncles (Yang et al., 2022).

Network pharmacology has found that SM can ameliorate DKD through mechanisms such as modulation of metabolism, improvement of renal hemodynamics, anti-oxidative stress, anti-inflammatory, anti-fibrotic, inhibition of great glucose-induced cellular damage, and improvement of autophagy (Pang et al., 2016; Wang et al., 2023).

Modern pharmacological studies have found that SM mainly contains two types of active ingredients: water-soluble phenolic acids (mainly salvianolic acid, Salvianolic acid A-E) and fat-soluble terpenoids (mainly Tanshinone IIA, Oleanolic acid, Ursolic acid), which act synergistically through multi-targets and multi-pathways, which is the pharmacological basis for the treatment of DKD (Kang et al., 2023; Cai et al., 2024; Shi et al., 2019; Lee et al., 2011).

Salvianolic acid B and Tanshinone IIA can synergistically downregulate 24-h urinary protein, serum creatinine, and urea levels in DKD rats, and improve lipid, steroid, and arachidonic acid metabolic pathways, thus exerting nephroprotective effects (Xu et al., 2024). Salvianolic acid A significantly inhibited the formation and accumulation of AGEs and attenuated glomerular injury by down-regulating RAGE expression, reducing oxidative stress and inflammatory responses, and improving endothelial cell autophagy (Hou et al., 2017).

Tanshinone IIA effectively delays the progression of DKD by regulating the activity of Txnip/NLRP3 inflammatory vesicles, inhibiting the pyroptosis pathway, and protecting microvascular endothelial cells (Wu et al., 2023). Oleanolic acid improves energy metabolism mainly through the AMPK/PGC-1α pathway and inhibits TLR4/NF-κB inflammatory signaling, reducing TGF-β1 and collagen deposition in renal tissues, thereby alleviating renal fibrosis and inflammatory response (Liu et al., 2022). Ursolic acid, on the other hand, significantly improved renal function indices such as blood urea nitrogen and creatinine levels and blocked renal structural damage in DKD rats through antioxidant and anti-inflammatory (Xu et al., 2018).

SM and its main active ingredients have a good pharmacological basis and application prospects in treating DKD through multi-target and multi-pathway modulation mechanisms, which supports its use as one of the candidates for clinical intervention.

This study is the first systematic evaluation and Meta-analysis focusing exclusively on the use of SM as a single agent in the treatment of DKD, which overcomes the problem of confounding effects caused by the combination of SM with other traditional Chinese medicines in previous studies. By including 21 randomized controlled trials, the clinical efficacy and safety data of different SM preparations in intervening DKD were systematically integrated. In this study, multiple aspects of renal function (Scr, BUN, 24 h urine protein, UAER), inflammatory factors (CRP, TNF-α, IL-6), and endothelial function indexes (ET-1, FDM) were comprehensively evaluated respectively, and the results showed that SM preparations had certain advantages in improving renal function, inhibiting the inflammatory response and enhancing vascular endothelial function, and had a good safety profile. The results of the study may provide research directions for subsequent high-quality clinical trials and provide scientific evidence for the evidence-based clinical application of SM preparations.

Firstly, despite conducting subgroup and sensitivity analyses, the main outcome indicators exhibited a high degree of heterogeneity. This heterogeneity likely arises from multiple sources, including: differences in the control group interventions adopted across the included studies; the use of varied types of SM preparations in the experimental groups, which inherently differ in their pharmacological activities, metabolic properties, and potentially their active component profiles due to variations in source materials, extraction methods, or manufacturing standards; and the fact that some studies did not report key baseline clinical characteristics such as patients’ DKD stage and duration of disease, potentially leading to inconsistent baseline levels. Secondly, insufficient reporting of intervention details in some studies, such as specific mechanisms of action, blinding procedures, trial registration information, and crucially, drug batch numbers, which made it difficult to assess the consistency, reproducibility, and potential sources of variation in the interventions. Thirdly, the predominance of studies sourced from Chinese language databases, often featuring single-center designs and small sample sizes, introduces risks of publication bias, language bias, and regional selection bias.

This also suggests that we should pay more attention to standardization and transparency in the design of future TCM studies. This includes comprehensive reporting of patient baseline information, detailed intervention protocols, which include formulation specifics and batch identifiers where possible, blinding methods, and registration details to enhance evidence reproducibility and clinical applicability. We also suggest that high-quality randomized controlled trials with multi-center, large samples, and long follow-up periods should be conducted in follow-up to further validate the efficacy and safety of SM preparations in the treatment of DKD.

## 5 Conclusion

Despite inherent challenges in botanical drug research, this study demonstrates compelling evidence that SM preparation is a extra ally in the fight against DKD. It effectively lowers indicators such as Scr, BUN, UAER, 24 h-utp, CRP, TNF-α, IL-6, and ET-1 while simultaneously raising FMD levels. This treatment not only enhances the clinical outcomes for patients but does so without introducing significant adverse effects when compared to standard control groups. These results illustrate that the thoughtful application of SM preparations can improve renal and vascular endothelial function as well as reduce harmful inflammatory factors in patients with DKD, making it a safe and effective choice for better health.

## Data Availability

The datasets presented in this study can be found in online repositories. The names of the repository/repositories and accession number(s) can be found in the article/supplementary material.
