# Supplementary material for: Effects and safety of Salvia miltiorrhiza on the improvement of renal function, inflammatory factors, and vascular endothelium in patients with diabetic kidney disease: a meta-analysis and systematic review
Source: Front Pharmacol. 2025 Sep 12;16:1556368. doi: 10.3389/fphar.2025.1556368 (PMC12464425; doi:10.3389/fphar.2025.1556368)
Supplement: Supplementary file 2 [file Table1.pdf]

Table 1 Search strategy table

Date of last search: May 31, 2025

Language: English, Chinese

Search strategy:

PubMed	
#1	"Diabetic Nephropathies"[Mesh] OR "Diabetic Nephropathy"[tiab] OR "Diabetic Kidney Disease"[tiab]
#2	(Diabetic Nephropathy [All Fields]) OR (Nephropathy, Diabetic[All Fields]) OR (Nephropathies, Diabetic[All Fields]) OR (Diabetic Nephropathies[All Fields]) OR (Diabetic Kidney Disease[All Fields]) OR (Diabetic Kidney Diseases[All Fields]) OR (Kidney Disease, Diabetic[All Fields]) OR (Kidney Diseases, Diabetic[All Fields]) OR (Diabetic Glomerulosclerosis[All Fields]) OR (Glomerulosclerosis,Diabetic[All Fields]) OR (Intracapillary Glomerulosclerosis[All Fields]) OR (Nodular Glomerulosclerosis[All Fields]) OR (Glomerulosclerosis,Nodular[All Fields]) OR (Kimmelstiel-Wilson Syndrome[All Fields]) OR (Kimmelstiel Wilson Syndrome[All Fields]) OR (Syndrome, Kimmelstiel -Wilson[All Fields]) OR (Kimmelstiel-Wilson Disease[All Fields]) OR (Kimmelstiel Wilson Disease[All Fields])
#3	#1 OR #2
#4	"Diabetic Nephropathies"[Mesh] OR "Diabetic Nephropathy"[tiab] OR "Diabetic Kidney Disease"[tiab]

#5	(Salvia miltiorrhizae[All Fields]) OR (Danshen[All Fields]) OR (Dan-Shen[All Fields]) OR (Dan Shen[All Fields]) OR (Chinese Salvia[All Fields]) OR (Chinese Salvias[All Fields]) OR (Salvia, Chinese[All Fields]) OR (Salvias, Chinese[All Fields]) OR (Tan Shen[All Fields])
#6	#4 OR #5
#7	(randomized controlled trial[pt] OR controlled clinical trial[pt] OR randomized[tiab] OR placebo[tiab] OR drug therapy[sh] OR randomly[tiab] OR trial[tiab] OR groups[tiab]) NOT (animals[mh] NOT humans[mh])
#8	#3 AND #6 AND #7
**Web of Science**	
#1	TS= (Diabetic Kidney Disease OR Diabetic Nephropathy OR Diabetic Nephropathies OR Diabetic Kidney Diseases OR Diabetic Glomerulosclerosis OR Intracapillary Glomerulosclerosis OR Nodular Glomerulosclerosis OR Kimmelstiel-Wilson Syndrome OR Kimmelstiel Wilson Syndrome OR Syndrome, Kimmelstiel-Wilson OR Kimmelstiel-Wilson Disease OR Kimmelstiel Wilson Disease)
#2	TS= (Salvia miltiorrhizae OR Danshen OR Dan-Shen OR Dan Shen OR Chinese Salvia OR Chinese Salvias OR Salvia, Chinese OR Salvias, Chinese OR Tan Shen)
#3	TS= (randomized controlled trial OR controlled clinical trial OR randomized OR placebo OR drug therapy OR randomly OR trial OR groups OR Intervention Study OR Clinical Study OR Intervention Study OR Clinical Study NOT animals NOT humans)

#4	#1 AND #2 AND #3
**Cochrane Library**	
#1	MeSH descriptor: [Diabetic Kidney Disease] explode all trees
#2	(Diabetic Nephropathy* OR Nephropathies, Diabetic* OR Nephropathy, Diabetic* OR Diabetic Nephropathies* OR Diabetic Kidney Diseases* OR Kidney Disease, Diabetic* OR Kidney Diseases, Diabetic* OR Diabetic Glomerulosclerosis* OR Glomerulosclerosis, Diabetic* OR Intracapillary lomerulosclerosis* OR Nodular Glomerulosclerosis* OR Glomerulosclerosis, Nodular* OR Kimmelstiel-Wilson Syndrome* OR Kimmelstiel WilsonSyndrome* OR Syndrome, Kimmelstiel-Wilson* OR Kimmelstiel-Wilson Disease* OR Kimmelstiel Wilson Disease*):ab,kw,ti
#3	#1 OR #2
#4	MeSH descriptor: [Salvia miltiorrhizae] explode all trees
#5	(Danshen* OR Dan-Shen* OR Dan Shen* OR Chinese Salvia* OR Chinese Salvias* OR Salvia, Chinese* OR Salvias, Chinese* OR Tan Seng*):ab,kw,ti
#6	#4 OR #5
#7	MeSH descriptor: [randomized controlled trial] explode all trees

#8	(randomized controlled trial* OR controlled clinical trial* OR randomized* OR placebo* OR drug therapy* OR randomly* OR trial* OR groups* OR Intervention Study* OR Clinical Study* OR Intervention Study* OR Clinical Study* NOT animals):ab,kw,ti
#9	#6 OR #7
#10	#3 AND #6 AND #9
**Elsevier Science Direct**	
#1	Diabetic Kidney Disease':ab,kw,ti OR 'Diabetic Nephropathy ':ab,kw,ti OR 'Nephropathy, Diabetic':ab,kw,ti OR 'Nephropathies, Diabetic':ab,kw,ti OR 'Diabetic Nephropathies':ab,kw,ti OR '(Diabetic Kidney Diseases':ab,kw,ti OR 'Kidney Disease, Diabetic':ab,kw,ti OR 'Kidney Diseases, Diabetic':ab,kw,ti OR 'DiabeticGlomerulosclerosis':ab,kw,ti OR 'Glomerulosclerosis, Diabetic':ab,kw,ti OR 'Intracapillary Glomerulosclerosis':ab,kw,ti OR 'Nodular Glomerulosclerosis':ab,kw,ti OR 'Glomerulosclerosis, Nodular':ab,kw,ti OR 'Kimmelstiel-Wilson Syndrome':ab,kw,ti OR 'Kimmelstiel Wilson Syndrome':ab,kw,ti OR 'Syndrome, Kimmelstiel-Wilson':ab,kw,ti OR 'Kimmelstiel-Wilson Disease':ab,kw,ti OR 'Kimmelstiel Wilson Disease '
#2	Salvia miltiorrhizae':ab,kw,ti OR 'Danshen':ab,kw,ti OR 'Dan-Shen':ab,kw,ti OR 'Dan Shen':ab,kw,ti OR 'Chinese Salvia':ab,kw,ti OR 'Chinese Salvias':ab,kw,ti OR 'Salvia, Chinese':ab,kw,ti OR 'Salvias, Chinese':ab,kw,ti OR 'Tan Seng':ab,kw,ti
#3	crossover procedure':de OR 'double-blind procedure':de OR 'randomized controlled trial':de OR 'single-blind procedure':de OR (random* OR factorial* OR crossover* OR cross NEXT/1 over* OR placebo* OR doubl* NEAR/1 blind* OR singl* NEAR/1 blind* OR assign* OR allocat* OR volunteer*) :de,ab,ti

#4	#1 AND #2 AND #3
**China National Knowledge Infrastructure (CNKI)**	
#1	(篇关摘=糖尿病肾脏病 + 糖尿病肾病 + 糖尿病性肾病 + 消渴肾病) AND (篇关摘=丹参 + 丹参制剂) AND (篇关摘=随机对照试验 + 临床试验)
**Wanfang database**	
#1	全部:(糖尿病肾脏病 + 糖尿病肾病 + 糖尿病性肾病 + 消渴肾病)and 全部:(丹参 + 丹参制剂)and 全部:(随机对照试验 + 临床试验)
**Database of Chinese sci-tech periodicals(VIP)**	
#1	篇关摘=糖尿病肾脏病+糖尿病肾病+糖尿病性肾病+消渴肾病 AND 篇关摘=丹参 AND 篇关摘=随机对照试验+临床试验

Table 2 Drug information

Study	Drug name	Manufacturer	China Drug Code	Production Batch Number	Mainly metabolite
2006 Hu Weifen	SM injection	Zhengda Qingchunbao Pharmaceutical Co., Ltd.	Z33020176	/	Over 40 types of phenolic compounds, including protocatechuic aldehyde, salvianolic acid B, rosmarinic acid, etc.
2007 Hu Meilan	SM injection	Zhengda Qingchunbao Pharmaceutical Co., Ltd.	Z33020176	/	Over 40 types of phenolic compounds, including protocatechuic aldehyde, salvianolic acid B, rosmarinic acid, etc.
2008 Liu Wenxing	Tanshinone IIA sodium sulfonate injection	SPH No.1 Biochemical & Pharmaceutical Co., Ltd.	H31022558	/	Sodium tanshinone IIA sulfonate
2011 Leng Kuangfeng	Tanshinone IIA sodium sulfonate injection	SPH No.1 Biochemical & Pharmaceutical Co., Ltd.	H31022558	100616	Sodium tanshinone IIA sulfonate

2012 Gong Baowen	Tanshinone IIA sodium sulfonate injection	SPH No.1 Biochemical & Pharmaceutical Co., Ltd.	H31022558	/	Sodium tanshinone IIA sulfonate
2014 Liu Weilin	Salvia divinorum polyphenate	Shanghai Greenvalley Pharmaceutical Co., Ltd.	Z20050249	/	High-purity phenolic salt mixtures, including magnesium salvianolate, magnesium salvia acid, sodium rosmarinic acid, potassium danshen acid, and potassium isodanshen acid, etc.
2014 Lu Weibo	Salvia divinorum polyphenate	Shanghai Greenvalley Pharmaceutical Co., Ltd.	Z20050249	/	High-purity phenolic salt mixtures, including magnesium salvianolate, magnesium salvia acid, sodium rosmarinic acid, potassium danshen acid, and potassium isodanshen acid, etc.
2015 Mayinu	Salvia divinorum polyphenate	Shanghai Greenvalley Pharmaceutical Co., Ltd.	Z20050249	20120831	High-purity phenolic salt mixtures, including magnesium salvianolate, magnesium salvia acid, sodium rosmarinic acid, potassium danshen acid, and potassium isodanshen acid, etc.

2015 Zhao Qi	Salvia divinorum polyphenate	Shanghai Greenvalley Pharmaceutical Co., Ltd.	Z20050249	13120426	High-purity phenolic salt mixtures, including magnesium salvianolate, magnesium salvia acid, sodium rosmarinic acid, potassium danshen acid, and potassium isodanshen acid, etc.
2016 Hu Bo	SM injection	Harbin Pharmaceutical Group Chinese Medicine No.2 Factory	Z23021510	/	Over 40 types of phenolic compounds, including protocatechuic aldehyde, salvianolic acid B, rosmarinic acid, etc.
2018 Chen Jun	Salvia divinorum polyphenate	Shanghai Greenvalley Pharmaceutical Co., Ltd.	Z20050249	/	High-purity phenolic salt mixtures, including magnesium salvianolate, magnesium salvia acid, sodium rosmarinic acid, potassium danshen acid, and potassium isodanshen acid, etc.

2018 Fu Liqun	Salvia divinorum polyphenate	Shanghai Greenvalley Pharmaceutical Co., Ltd.	Z20050248	1509081、1604122	High-purity phenolic salt mixtures, including magnesium salvianolate, magnesium salvia acid, sodium rosmarinic acid, potassium danshen acid, and potassium isodanshen acid, etc.
2018 Qiao Honggao	Tanshinone IIA sodium sulfonate injection	SPH No.1 Biochemical & Pharmaceutical Co., Ltd.	H31022558	/	Sodium tanshinone IIA sulfonate
2018 Wang Linlin	Salvia divinorum polyphenate	Shanghai Greenvalley Pharmaceutical Co., Ltd.	Z20050249	13082821	High-purity phenolic salt mixtures, including magnesium salvianolate, magnesium salvia acid, sodium rosmarinic acid, potassium danshen acid, and potassium isodanshen acid, etc.
2018 Zheng Jun	SM injection	Jilin Huinan Huifa Pharmaceutical Co., Ltd.	Z22020704	/	Over 40 types of phenolic compounds, including protocatechuic aldehyde, salvianolic acid B, rosmarinic acid, etc.

2019 He Wanxia	Salvia divinorum polyphenate	Shanghai Greenvalley Pharmaceutical Co., Ltd.	Z20050249	/	High-purity phenolic salt mixtures, including magnesium salvianolate, magnesium salvia acid, sodium rosmarinic acid, potassium danshen acid, and potassium isodanshen acid, etc.
2020 Hong Lijuan	Salvia divinorum polyphenate	Tianjin Tasly Pride Pharmaceutical Co., Ltd.	Z20110011	/	High-purity phenolic acid salt mixtures, including salvianolic acid B, rosmarinic acid, lithospermic acid, danfen acid Y, and other water-soluble phenolic acids.
2020 Li Zhaoliang	SM injection	CHIA TAI Tianqing Pharmaceutical Group Co., Ltd.	Z32021228	/	Over 40 types of phenolic compounds, including protocatechuic aldehyde, salvianolic acid B, rosmarinic acid, etc.
2021 He Zhiting	Tanshinone IIA sodium sulfonate injection	SPH No.1 Biochemical & Pharmaceutical Co., Ltd.	H31022558	/	Sodium tanshinone IIA sulfonate

2021 Wang Han	Salvia divinorum polyphenate	Shanghai Greenvalley Pharmaceutical Co., Ltd.	Z20050247	/	High-purity phenolic salt mixtures, including magnesium salvianolate, magnesium salvia acid, sodium rosmarinic acid, potassium danshen acid, and potassium isodanshen acid, etc.
2025 ZhaoJing	Salvia divinorum polyphenate	Shanghai Greenvalley Pharmaceutical Co., Ltd.	Z20050249	/	High-purity phenolic salt mixtures, including magnesium salvianolate, magnesium salvia acid, sodium rosmarinic acid, potassium danshen acid, and potassium isodanshen acid, etc.

Table 3 Study character

study	Sample size	Age(y)	Gender(m:f)	DKD duration(y)	DKD staging	treatment duration(w)
	E/C	E/C	E/C	E/C		
2006 Hu Weifen	30/28	56±10/58.9±9.9	16:14/18:10	5.6±1.7/5.8±2.0	III	3
2007 Hu Meilan	32/30	52.5(40~68)/51.3(35~66)	19:13/18:12	4.8(0.75~8)/4.2(0.9~7)	III	4
2008 Liu Wenxing	36/36	47.5±10.6	30:42	/	III	4
2011 Leng Kuangfeng	47/47	53±4	25:22/25:22	12±2	/	52
2012 Gong Baowen	39/39	51.2±6.9/50.3±7.3	19:20/20:19	51.2±6.9/8.5±4.3	III	2
2014 Liu Weilin	30/30	65.12±9.83/65.40±9.60	14:16/17:13	/	III	2
2014 Lu Weibo	33/31	56.16±9.09/54.70±10.26	16:17/16:15	/	III	2
2015 Mayinu	59/59	65.3±7.1/63.7±7.4	29:30/27:32	/	III	2
2015 Zhao Qi	45/45	63.4±9.8/7.1±2.4	24:21/24:21	7.1±2.4/7.3±1.4	/	4
2016 Hu Bo	184/184	59.14±9.01/58.67±8.42	100：84/96:88	/	/	4

2018 Chen Jun	60/60	68.45±6.45/68.82±8.63	39:21/38:22	6.54±2.64/68.45±8.35	/	12
2018 Fu Liqun	38/38	56.22±9.67/52.15±10.55	22:16/18:20	52.15±10.55/6.04±2.03	/	2
2018 Qiao Honggao	60/60	49.24±6.21/45.32±6.26	36:24/33:27	5.0±1.7/5.4±1.5	/	2
2018 Wang Linlin	45/45	53.74±6.54/54.74±6.26	28:17/30:15	5.32±1.43/5.56±1.62	/	2
2018 Zheng Jun	42/42	62.14±11.83/61.46±12.04	29:13/26:16	3.99±0.74/4.04±0.72	/	4
2019 He Wanxia	26/26	55.97±10.63/56.21±10.89	16:10/17:9	11.62±6.13/11.68±6.46	V	2
2020 Hong Lijuan	25/25	48.40±6.78/48.66±6.52	13:12/14:11	11.69±3.03/11.55±3.12	III	2
2020 Li Zhaoliang	40/40	50.13±9.82	45：35	4.3±1.2	≤III	8
2021 He Zhiting	41/41	49.11±2.19/49.22±2.22	21:20/18:23	/	/	4
2021 Wang Han	31/31	56.12±8.26/58.12±8.45	16:15/15:16	5.12±1.23/5.42±1.26	/	2
2025 ZhaoJing	45/45	60.51±9.07/62.13±9.32	27:18/26:19	8.53±1.27/9.51±1.42	/	2

Tablet 4 Study interventions

study	intervention	
	treatment group	control group
2006 Hu Weifen	SM injection 50ml I.v.gtt qd+perindopril tablet 4mg PO qd	perindopril tablet 4mg PO qd
2007 Hu Meilan	SM injection 20ml I.v.gtt qd+Shenmai injection 30 IV qd	Shenmai injection 30 IV qd
2008 Liu Wenxing	Tanshinone IIA sodium sulfonate injection 60mg I.v.gtt qd+Irbesartan tablet 150mg PO qd	Irbesartan tablet 150mg PO qd
2011 Leng Kuangfeng	Tanshinone IIA sodium sulfonate injection 60mg I.v.gtt qd+Benadryl tablet 10mg PO qd	Benazapril tablet 10mg PO qd
2012 Gong Baowen	Tanshinone IIA sodium sulfonate injection 40mg I.v.gtt qd+Prostaglandin injection 10μg IV	Prostaglandin injection 10μg IV
2014 Liu Weilin	Salvia divinorum polyphenate 200mg I.v.gtt qd	routine treatment
2014 Lu Weibo	Salvia divinorum polyphenate 200mg I.v.gtt qd+insulin	insulin

2015 Mayinu	Salvia divinorum polyphenate 200mg I.v.gtt qd	routine treatment
2015 Zhao Qi	Salvia divinorum polyphenate 200mg I.v.gtt qd	routine treatment
2016 Hu Bo	SM injection 400mg I.v.gtt qd+Benazapril tablet 10mg PO qd	Benazapril tablet 10mg PO qd
2018 Chen Jun	Salvia divinorum polyphenate 200mg I.v.gtt qd+Chlorosartan potassium tablet 50mg PO qd	Chlorosartan potassium tablet 50mg PO qd
2018 Fu Liqun	Salvia divinorum polyphenate 100mg I.v.gtt qd+Irbesartan capsule 150mg PO qd	Irbesartan capsule 150mg PO qd
2018 Qiao Honggao	Tanshinone IIA sodium sulfonate injection 40mg I.v.gtt qd+Mentholated insulin 30 injection IH bid	Mentholated insulin 30 injection IH bid
2018 Wang Linlin	Salvia divinorum polyphenate 200mg I.v.gtt qd	Chlorosartan potassium tablet 50mg PO qd
2018 Zheng Jun	SM injection 30ml I.v.gtt qd+Alfacalcitol 0.25μg PO qd	Alfacalcitol 0.25μg PO qd
2019 He Wanxia	Salvia divinorum polyphenate 200mg I.v.gtt qd+Dialysis 4h/times tiw	Dialysis 4h/times tiw
2020 Hong Lijuan	Salvia divinorum polyphenate 200mg I.v.gtt qd	routine treatment

2020 Li Zhaoliang	SM injection 10ml I.v.gtt qd+Valsartan 80mg qd	Valsartan 80mg qd
2021 He Zhiting	Tanshinone IIA sodium sulfonate injection 40~80 mg I.v.qd++Dialysis biw~tiw	Dialysis biw~tiw
2021 Wang Han	Salvia divinorum polyphenate 200mg I.v.gtt qd+Irbesartan tablet 150mg PO qd	Irbesartan tablet 150mg PO qd
2025 ZhaoJing	Salvia divinorum polyphenate 200mg I.v.gtt qd+Valsartan Capsules 80mg qd	Valsartan Capsules 80mg qd

Table 5 GRADE quality assessment table

study	study type	Risk of bias	Imprecision	Inconsistency	Indirectness	Publication bias	Overall quality assessment
2006 Hu Weifen	RCT	Failure to mention distribution concealment or blinding, downgraded by 1 level	Confidence interval unstable, downgraded by 1 level	Internal consistency of results	No apparent indirect	No explicit reference to registration information, but published in core journals with standardised sources	low
2007 Hu Meilan	RCT	Failure to mention distribution concealment or blinding, downgraded by 1 level	Confidence interval unstable, downgraded by 1 level	Internal consistency of results	No apparent indirect	No explicit reference to registration information, but published in core journals with standardised sources	low
2008 Liu Wenxing	RCT	Failure to mention distribution concealment or blinding, downgraded by 1 level	Stability of the confidence interval	Internal consistency of results	No apparent indirect	No explicit reference to registration information, but published in core journals with standardised sources	middle
2011 Leng Kuangfeng	RCT	Failure to mention distribution concealment or blinding, downgraded by 1 level	Stability of the confidence interval	Internal consistency of results	No apparent indirect	No explicit reference to registration information, but published in core journals with standardised sources	middle
2012 Gong Baowen	RCT	Failure to mention distribution concealment or blinding, downgraded by 1 level	Stability of the confidence interval	Internal consistency of results	No apparent indirect	No explicit reference to registration information, but published in core journals with standardised sources	middle

2014 Liu Weilin	RCT	Failure to mention distribution concealment or blinding, downgraded by 1 level	Stability of the confidence interval	Internal consistency of results	No apparent indirect	No explicit reference to registration information, but published in core journals with standardised sources	middle
2014 Lu Weibo	RCT	Failure to mention distribution concealment or blinding, downgraded by 1 level	Stability of the confidence interval	Internal consistency of results	No apparent indirect	No explicit reference to registration information, but published in core journals with standardised sources	middle
2015 Mayinu	RCT	Failure to mention distribution concealment or blinding, downgraded by 1 level	Stability of the confidence interval	Internal consistency of results	No apparent indirect	No explicit reference to registration information, but published in core journals with standardised sources	middle
2015 Zhao Qi	RCT	Failure to mention distribution concealment or blinding, downgraded by 1 level	Confidence interval unstable, downgraded by 1 level	Internal consistency of results	No apparent indirect	No explicit reference to registration information, but published in core journals with standardised sources	low
2016 Hu Bo	RCT	Failure to mention distribution concealment or blinding, downgraded by 1 level	Stability of the confidence interval	Internal consistency of results	No apparent indirect	No explicit reference to registration information, but published in core journals with standardised sources	middle
2018 Chen Jun	RCT	Failure to mention distribution concealment or blinding, downgraded by 1 level	Stability of the confidence interval	Internal consistency of results	No apparent indirect	No explicit reference to registration information, but published in core journals with standardised sources	middle

2018 Fu Liqun	RCT	Failure to mention distribution concealment or blinding, downgraded by 1 level	Confidence interval unstable, downgraded by 1 level	Internal consistency of results	No apparent indirect	No explicit reference to registration information, but published in core journals with standardised sources	low
2018 Qiao Honggao	RCT	Failure to mention distribution concealment or blinding, downgraded by 1 level	Stability of the confidence interval	Internal consistency of results	No apparent indirect	No explicit reference to registration information, but published in core journals with standardised sources	middle
2018 Wang Linlin	RCT	Failure to mention distribution concealment or blinding, downgraded by 1 level	Stability of the confidence interval	Internal consistency of results	No apparent indirect	No explicit reference to registration information, but published in core journals with standardised sources	middle
2018 Zheng Jun	RCT	Failure to mention distribution concealment or blinding, downgraded by 1 level	Stability of the confidence interval	Internal consistency of results	No apparent indirect	No explicit reference to registration information, but published in core journals with standardised sources	middle
2019 He Wanxia	RCT	Failure to mention distribution concealment or blinding, downgraded by 1 level	Stability of the confidence interval	Internal consistency of results	No apparent indirect	No explicit reference to registration information, but published in core journals with standardised sources	middle
2020 Hong Lijuan	RCT	Failure to mention distribution concealment or blinding, downgraded by 1 level	Stability of the confidence interval	Internal consistency of results	No apparent indirect	Failure to explicitly mention registration information downgraded by 1 level	low

2020 Li Zhaoliang	RCT	Failure to mention distribution concealment or blinding, downgraded by 1 level	Confidence interval unstable, downgraded by 1 level	Internal consistency of results	No apparent indirect	No explicit reference to registration information, but published in core journals with standardised sources	low
2021 He Zhiting	RCT	Failure to mention distribution concealment or blinding, downgraded by 1 level	Confidence interval unstable, downgraded by 1 level	Internal consistency of results	No apparent indirect	Failure to explicitly mention registration information downgraded by 1 level	extremely low
2021 Wang Han	RCT	Failure to mention distribution concealment or blinding, downgraded by 1 level	Stability of the confidence interval	Internal consistency of results	No apparent indirect	Failure to explicitly mention registration information downgraded by 1 level	low
2025 ZhaoJing	RCT	Failure to mention distribution concealment or blinding, downgraded by 1 level	Stability of the confidence interval	Internal consistency of results	No apparent indirect	Failure to explicitly mention registration information downgraded by 1 level	low

Table 6 Adverse events

year	study	adverse events	
		E	C
2008	Liu Wenxing	1 case of mild dizziness and thirst	No report
2012	Gong Baowen	1 case of palpitations and chest tightness during infusion,	No report
2014	Liu Weilin	No report	No report
2015	Zhao Qi	No report	No report
2016	Hu Bo	6 cases of elevated blood potassium	19 cases of levated blood potassium
2018	Fu Liqun	No report	No report
2018	Qiao Honggao	1 case of hypoglycaemia, 1 case of nausea, 1 case of abdominal distension	2 cases of hypoglycaemia, 2 nausea, 1 bloating
2018	Wang Linlin	2 cases of mild itching and local pain at the injection site.	2 cases of cough, 2 cases of headache and dizziness.

2018	Zheng Jun	3 cases of fever, 1 case of diarrhoea, 2 cases of skin rash	3 cases of fever, 1 case of rash.
2020	Hong Lijuan	2 cases of dizziness	4 cases of dizziness and headache
